# Fabrication of Microparticles with Front–Back Asymmetric Shapes Using Anisotropic Gelation

**DOI:** 10.3390/mi12091121

**Published:** 2021-09-17

**Authors:** Dongkyu Lee, Hiroyuki Kitahata, Hiroaki Ito

**Affiliations:** Department of Physics, Chiba University, Chiba 263-8522, Japan; caxa6130@chiba-u.jp (D.L.); kitahata@chiba-u.jp (H.K.)

**Keywords:** droplet-based microfluidics, droplet fusion, gelation, non-spherical shape, front–back asymmetry

## Abstract

Droplet-based microfluidics is a powerful tool for producing monodispersed micrometer-sized droplets with controlled sizes and shapes; thus, it has been widely applied in diverse fields from fundamental science to industries. Toward a simpler method for fabricating microparticles with front–back asymmetry in their shapes, we studied anisotropic gelation of alginate droplets, which occurs inside a flow-focusing microfluidic device. In the proposed method, sodium alginate (NaAlg) aqueous phase fused with a calcium chloride (${\mathrm{CaCl}}_{2}$) emulsion dispersed in the organic phase just before the aqueous phase breaks up into the droplets. The fused droplet with a front–back asymmetric shape was generated, and the asymmetric shape was kept after geometrical confinement by a narrow microchannel was removed. The shape of the fused droplet depended on the size of prefused NaAlg aqueous phase and a ${\mathrm{CaCl}}_{2}$ emulsion, and the front–back asymmetry appeared in the case of the smaller emulsion size. The analysis of the velocity field inside and around the droplet revealed that the stagnation point at the tip of the aqueous phase also played an important role. The proposed mechanism will be potentially applicable as a novel fabrication technique of microparticles with asymmetric shapes.

## 1. Introduction

Droplet-based microfluidics has been widely applied in diverse fields from fundamental science to industries [1,2]. Chemical analyses [3,4] and biological assays [5,6] are successful examples of the droplet-based microfluidics since it enables high-throughput and high-precision treatments of a tiny amount of reagents. In these processes, the micrometer-sized droplets are used as isolated “microreactors” in a confined environment, and various microfluidic techniques such as sorting [7,8,9,10], mixing [11,12], and concentrating [13,14] serve the powerful platforms to manipulate the droplets in an automated and sophisticated manner. In addition, the droplet-based microfluidics is also useful for producing the microparticles with uniform sizes and shapes in the fields of drug delivery [15,16], optical devices [17,18], etc. In the fabrication of microparticles, droplets are used as templates with controlled shapes and sizes [19,20,21,22].

In the droplet-based microfluidics, immiscible different fluids, typically an aqueous solution and an organic solution, produce the dispersed and continuous phases after they come into contact with each other at a junction designed in a microchannel. In order to produce micrometer-sized droplets under passive laminar flow, various geometries of the junctions, such as T-junction [23,24,25], co-flow [26,27], and flow-focusing [28,29], have been proposed in the past decades. At the junctions, one fluid phase spontaneously breaks up into monodisperse droplets by the interfacial tensions and viscous shear stresses [27,29,30]. After the break up into the droplets, they take the form of spheres, circular disks, plugs, and threads depending on the geometrical confinement [19,20,31,32], i.e., the width and height, of the microchannel. These shapes are highly symmetric in geometry because the interfacial tension becomes a dominant factor in determining the shape of microdroplets due to the high surface-to-volume ratio on a microscopic scale.

For the fabrication of non-uniform microparticles, multi-emulsions, which are the droplets encapsulating several smaller droplets, made by T-junction, co-flowing, or flow-focusing devices [33,34] are often used as templates. Core-shell double emulsions, which are the droplets encapsulating a single smaller droplet of an immiscible liquid in each one of them, are the most typical case of this kind. They are spherical in their shapes and non-uniform in their inner structures due to the minimization of the contact of the outer shell with both the channel wall and the other immiscible liquid phase. Janus droplets are another class of non-uniform droplets, which have two distinct compositions and/or surface features at one half and the other half in a single droplet. One approach to producing Janus droplets by using droplet microfluidics is dripping (typically flow-focusing) the parallel two-phase flow of two different polymer solutions [35,36,37,38,39]. The Janus droplets obtained inside a microchannel are used as the templates for fabricating solidified Janus particles.

The ideas with two different immiscible phases are also adopted to fabricate non-spherical microparticles. Using two immiscible polymer solutions that exhibit phase separation, not only core-shell structures but also dumbbell-like and partially wetted asymmetric (termed “acorn-shaped”) structures can be formed through coalescence of the two droplets [40]. The stable morphology of the coalesced droplets can be tuned by the interfacial tensions between the two solutions and the dispersion medium; thus, such asymmetric double droplets can be used as templates for synthesizing highly functionalized microparticles with spatial asymmetry.

While the generated droplets are typically stabilized by the self-assembly of surfactant molecules at the interface, their shapes and sizes are metastable. In order to fabricate stable microparticles with controlled shapes and sizes through the droplet-based microfluidics, further decrease in surface area by interfacial tension, coalescence, and Ostwald ripening needs to be avoided. In order to permanently preserve droplet morphology, gelation in a microchannel has been adopted by using aqueous polymer droplets of poly(ethylene glycol) (PEG) [41], alginate [32,42,43], poly(lactic-co-glycolic acid) (PLGA) [44], etc. The gelation of the polymer droplets inside a microchannel is performed by photo-crosslinking by ultraviolet (UV) irradiation or chemical-crosslinking. The shapes of the obtained microparticles are in most cases spherical, ellipsoidal, or discoidal, all of which are highly axisymmetric [19,20,32,45]. Hayakawa et al. reported the asymmetric propeller-shaped agarose microparticles by dissolving sacrifice parts made of calcium alginate gel [46,47]. Deng et al. developed a strategy to fabricate rod-like, maraca-like, and pear-like particles with the diameter of several hundred micrometers using oil droplet templates generated with a co-flow device [48]. In this context of fabricating asymmetric microparticles by droplet microfluidics, there is still plenty of room to explore the simpler methods for fabricating microparticles with more complex shapes. In particular, the reduction in synthesizing steps and invention of a novel mechanism for producing microparticles with desired morphologies can contribute to various applications such as micromachines and biomedical drug carriers.

In this paper, we report a microfluidic technique to generate microparticles with front–back asymmetry in their shapes. For this purpose, we employed the rapid gelation of alginate droplets inside a flow-focusing microfluidic channel and studied appropriate conditions for anisotropic gelation. The key mechanism of the proposed method is droplet fusion during a dripping regime at around the channel junction in which the flow profile around the alginate droplet can induce the anisotropic gelation. First, we checked the dependence of the frequency and radius of the droplets on the driving pressures at the inlets for continuous and dispersed phases in the proposed microchannel. Next, we investigated the preferred droplet size for the formation of the microparticles with front–back asymmetry in their shapes. Finally, we discuss the detailed mechanism of anisotropic gelation through the analysis of a flow field around the droplet.

## 2. Materials and Methods

### 2.1. Sample Preparation

${\mathrm{CaCl}}_{2}$ (Kanto Chemical, Tokyo, Japan) aqueous solution was prepared at 2 M, and 80 μL of this solution was added into the 1 mL organic solution of mineral oil (Sigma Aldrich, St. Louis, MO, USA) containing 0.1% (*v/v*) sorbitan monooleate (Span-80) (Sigma Aldrich, St. Louis, MO, USA) as a surfactant. The ${\mathrm{CaCl}}_{2}$ aqueous solution and the organic solution were stirred by a vortex mixer for 10 s followed by sonication at 40 kHz for 10 s, resulting in the formation of emulsified ${\mathrm{CaCl}}_{2}$ aqueous droplets with various sizes, typically ranging from sub-micrometer to tens of micrometer. Sodium alginate (NaAlg) (Sodium Alginate 80–120, FUJIFILM Wako Pure Chemical, Osaka, Japan) aqueous solution was prepared at 3% (*w/w*) as the dispersed phase. The viscosities of the 2 M ${\mathrm{CaCl}}_{2}$ aqueous solution, the mineral oil with 0.1% Span-80, and the 3% NaAlg aqueous solution measured with the viscometer (SV-10A, A & D, Tokyo, Japan) were 1.85±0.03mPa·s, 22.18±0.24mPa·s, and 440.2±1.9mPa·s, respectively. For the experiment of particle image velocimetry (PIV) analysis, 0.3% (*v/v*) Span-80 was used as a surfactant in the organic phase, and polystyrene beads with the diameter 2 μm (Polysciences, Warrington, PA, USA) were added into both the organic and aqueous solutions.

### 2.2. Microfluidic Flow-Focusing Device

**Figure 1:**
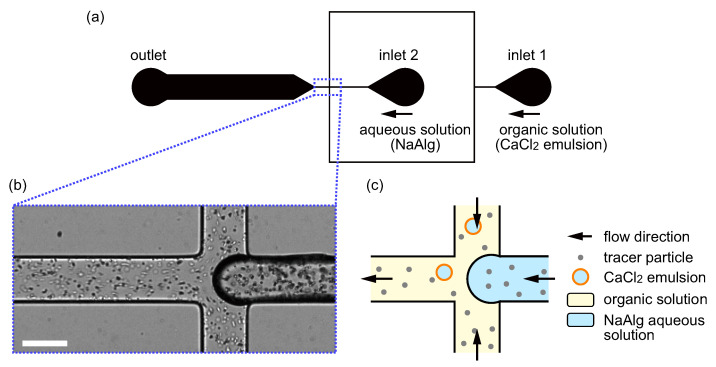
Experimental system for the production of droplets with an asymmetric shape. (**a**) Schematic diagram of the channel design. An organic solution containing ${\mathrm{CaCl}}_{2}$ emulsions and an aqueous solution of NaAlg contact with each other at the junction indicated by the blue dashed rectangle. Both the channel height and width around the junction are 50μm. (**b**) Microscopic image of the junction. Scale bar, 50μm. (**c**) Schematic illustration of the flow at the junction.

Figure 1a shows the design of the microfluidic flow-focusing device for the production of droplets with an asymmetric shape. The microchannels were prepared by standard soft lithography [49]. The microchannel was made of poly(dimethylsiloxane) (PDMS) (SILPOT 184, Dow Corning Toray, Tokyo, Japan) bonded onto a glass substrate. The master mold for replicating PDMS chips was prepared by negative photoresist (SU-8 3025, KAYAKU Advanced Materials, Inc., Westborough, MA, USA) via standard photolithography. The height of the channel was homogeneously set at 50μm. PDMS and the curing agent were mixed at a ratio 9:1, and the mixture was completely degassed under vacuum. Then, the mixture was poured onto the mold and was baked at 90 ${}^{\circ }C$ for 40 min. Afterward, the mold was removed, and holes were punched for the inlets and outlet. The surfaces of the PDMS chip and the glass substrate were treated by a corona charger (BD-20, Electro-Technic Products, Chicago, IL, USA) and were bonded to form a microchannel. Finally, the microfluidic chip was heated at 95 ${}^{\circ }C$ for 10 min by a hot plate (ND-1A, AsOne, Osaka, Japan).

### 2.3. Flow Experiment and Observation

The organic and aqueous solutions were, respectively, introduced from the inlet 1 and inlet 2 of the microfluidic flow-focusing device, as shown in Figure 1a, via poly(tetrafluoroethylene) (PTFE) tubes and silicone tubes. A pneumatic pump (MFCS${}^{\mathrm{TM}}$-EZ, Fluigent, Le Kremlin-Bicêtre, France) was used to control the driving pressures at the inlets 1 and 2. At the junction part of the microchannel, where a magnified microscopic image and a schematic illustration are shown in Figure 1b,c, respectively, the aqueous solution was dispersed into the organic solution, resulting in the formation of droplets. In order to observe droplet generation and gelation, the microfluidic chip was mounted on an inverted microscope (IX71, Olympus, Tokyo, Japan) equipped with a ×10 objective lens (NA=0.30, UPlanFLN, Olympus, Tokyo, Japan) and a high-speed camera (IDP-Express R2000, Photron, Tokyo, Japan). The camera view was set around the junction, and the recording frequency and exposure times were set at 1000 Hz and 0.5 ms, respectively. The typical Reynolds number for the flow of the organic solution, $Re∼6.47\times 10^{-3}$, was small enough to satisfy the condition for the laminar flow, Re≪1. Under typical conditions, the percentage of the unfused and fused droplets were ∼77.4% (326/421) and ∼22.6% (95/421), respectively. The percentage of the obtained front–back asymmetric droplets was ∼7.8% (33/421).

## 3. Results

### 3.1. Droplet Generation

**Figure 2:**
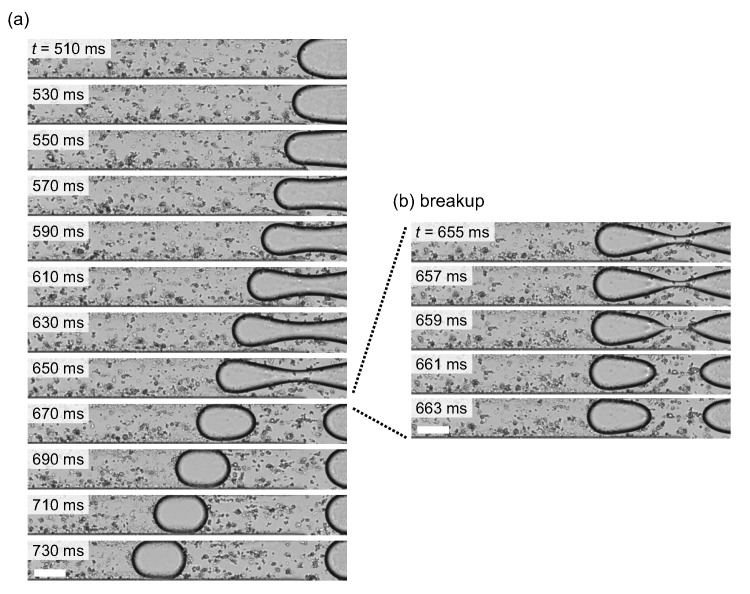
(**a**) Snapshots of the generation of a droplet with symmetric shape. t=0ms was set at a timing when the NaAlg aqueous solution retreated the most during periodic dripping. (**b**) Detailed view of the split of the droplet from the NaAlg aqueous phase. All scale bars, 50μm. The video is available in Supplementary Materials (Video S1).

For appropriate pressure inputs from the inlets 1 and 2, the NaAlg aqueous droplets were periodically generated at the junction in a microchannel (dripping regime) [36]. Figure 2a shows the typical snapshots of the droplet generation within a single cycle. t=0ms was set at a timing when the NaAlg aqueous solution retreated the most during periodic dripping. After slow dilation of the aqueous phase, the neck part was formed in the middle of the aqueous region, and finally the tip was pinched off. Figure 2b shows the detailed view of the split of the droplet from the NaAlg aqueous phase, where the thinning process developed within several milliseconds. The generated droplet rapidly changed its shape into the front–back symmetric shape as observed during t=690–710ms in Figure 2a. At t=730ms, the droplet slightly deformed into a “streamline” shape due to shear stresses and pressure gradients in the confined laminar flow, but the shape of the front interface was still round, which is a sign for the surface-area minimization dominated by the interfacial tension.

**Figure 3:**
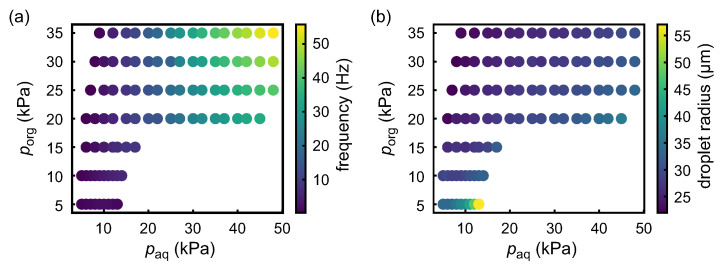
(**a**) Frequency of droplet generation as a function of the inlet gauge pressure for the organic solution (inlet 1) $p_{\mathrm{org}}$ and that for the aqueous solution (inlet 2) $p_{\mathrm{aq}}$. Data points are plotted only in regions where the droplets were generated. (**b**) Radius of the generated droplet as a function of the inlet pressure for the organic solution (inlet 1) $p_{\mathrm{org}}$ and that for the aqueous solution (inlet 2) $p_{\mathrm{aq}}$.

Figure 3 shows the properties of the droplet generation in the proposed microchannel depending on each inlet pressure, which is proportional to the flow rate in the connected branch of the microchannel. We checked the frequency of periodic droplet generation and the radius of the generated monodispersed droplet as a function of the inlet gauge pressure for the organic solution (inlet 1) $p_{\mathrm{org}}$ and that for the aqueous solution (inlet 2) $p_{\mathrm{aq}}$. As shown in the diagrams, the generation frequency was positively correlated with both the pressure inputs at the inlets 1, $p_{\mathrm{org}}$, and 2, $p_{\mathrm{aq}}$ (Figure 3a), while the droplet radius was negatively correlated with $p_{\mathrm{org}}$ and positively correlated with $p_{\mathrm{aq}}$. Based on this result, the generation frequency and the droplet radius can be independently tuned. In the following experiments, we chose a sufficiently small frequency to keep the periodically generated droplets isolated from each other, and varied the radius of the generated NaAlg aqueous droplets in order to investigate the appropriate condition to form asymmetric droplets.

### 3.2. Droplets Generation Coupled with Anisotropic Gelation

**Figure 4:**
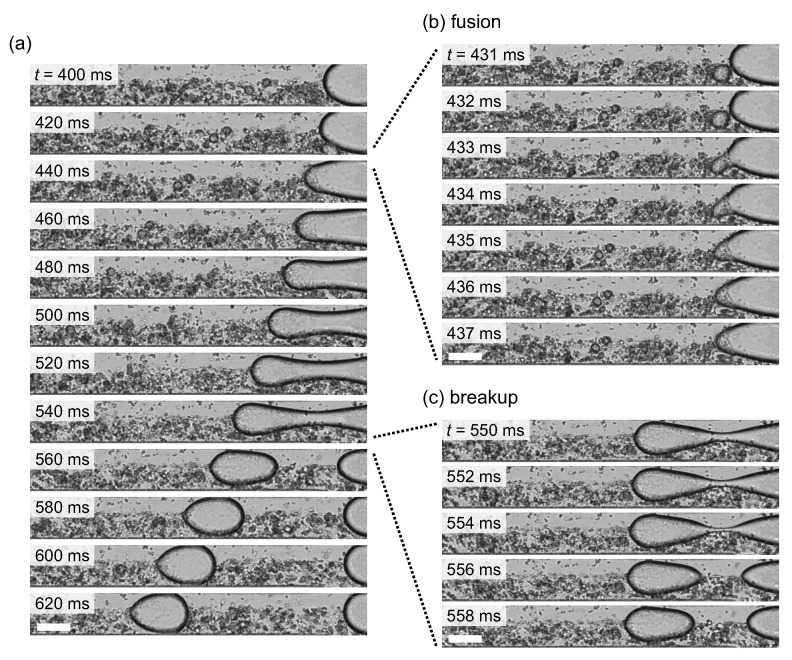
(**a**) Snapshots of the generation of a droplet with an asymmetric shape. t=0ms was set at the timing when the NaAlg aqueous phase retreated the most during periodic dripping. (**b**) Detailed view of the fusion with a small ${\mathrm{CaCl}}_{2}$ emulsion. (**c**) Detailed view of the split of the droplet from the NaAlg aqueous phase. All scale bars, 50μm. The video is available in Supplementary materials (Video S2).

**Figure 5:**
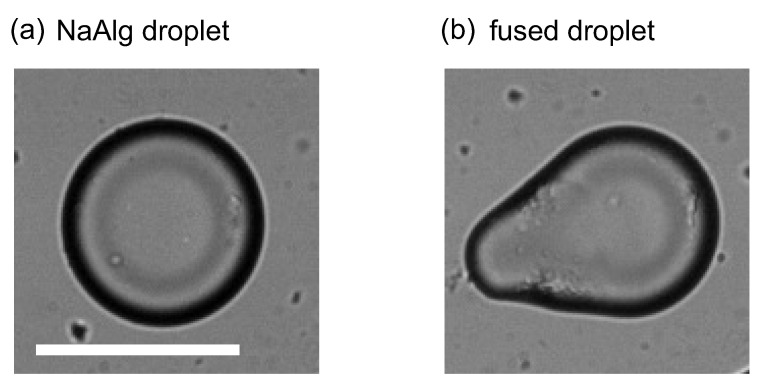
Snapshots of (**a**) a NaAlg aqueous droplet and (**b**) a fused droplet after the droplets entered the wide part of the microchannel. Scale bar, 50μm. The videos corresponding to (**a**,**b**) are available in Supplementary materials (Video S3 and Video S4, respectively).

Next, toward the fabrication of the droplets with an asymmetric shape, we introduced the organic solution in which the various micrometer-sized ${\mathrm{CaCl}}_{2}$ emulsions, i.e., ${\mathrm{CaCl}}_{2}$ small aqueous droplets, were dispersed from the inlet 1. At the junction of the microchannel, the ${\mathrm{CaCl}}_{2}$ emulsions coalesced with the NaAlg aqueous phase and stochastically fused into the NaAlg aqueous phase, which resulted in the gelation of alginate into calcium alginate hydrogel. Figure 4a shows the typical snapshots of the droplet generation during which a ${\mathrm{CaCl}}_{2}$ emulsion fused into the NaAlg aqueous phase. As shown in the detailed view of the fusion, the fused location was typically around the tip of the NaAlg aqueous phase, and the tip shape became sharper as the rapid gelation proceeded. Subsequently, the NaAlg aqueous phase was gradually dilated and pinched off, and an anisotropically gelated droplet was formed (Figure 4c). After the formation of the droplet, the droplet kept its asymmetric shape and went away along the main flow. Figure 5 shows typical snapshots of a NaAlg aqueous droplet and a fused droplet after the droplets entered the wide part of the microchannel, where the droplets were free from spatial confinement under the narrow microchannel. While the NaAlg aqueous droplet took a circular shape without fusing with a ${\mathrm{CaCl}}_{2}$ emulsion (Figure 5a), the droplet fused with ${\mathrm{CaCl}}_{2}$ emulsion kept its asymmetric shape without confinement (Figure 5b), suggesting its potential to be a template for the fabrication of a solidified microparticle.

**Figure 6:**
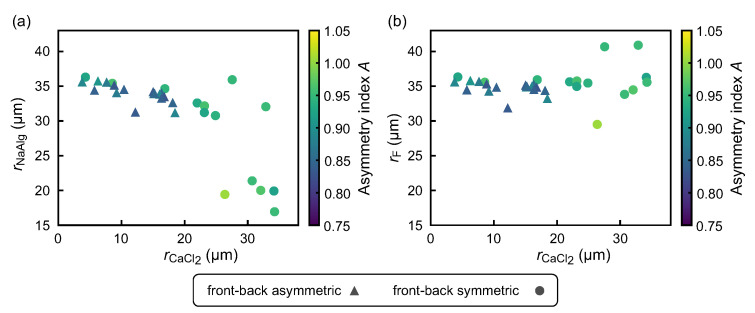
(**a**) Scatter plot of the radii of a NaAlg aqueous droplet $r_{\mathrm{NaAlg}}$ and a ${\mathrm{CaCl}}_{2}$ emulsion *r*CaCl_2_ measured before fusion. (**b**) Scatter plot of the radii of a fused droplet $r_{F}$ and the corresponding prefused ${\mathrm{CaCl}}_{2}$ emulsion $r_{\mathrm{CaCl}}2$. The shape of a fused droplet is indicated by circles (front–back symmetric) or triangles (front–back asymmetric). Color code indicates the asymmetry index *A*. Only the data points for which the droplet fusion occurred are plotted.

In rapid gelation after the fusion of a NaAlg droplet and a ${\mathrm{CaCl}}_{2}$ emulsion, the prefused amounts of these two species can significantly contribute to determining the resultant shape of the postfused droplet. Figure 6 shows the dependence of the morphologies of the postfused droplets on the sizes of prefused NaAlg aqueous droplets and ${\mathrm{CaCl}}_{2}$ emulsions. Since fusion occurred just before the breakup of the NaAlg aqueous phase, the amount of the prefused NaAlg aqueous solution could not be measured directly. Thus, we estimated the volume of a prefused NaAlg aqueous droplet by subtracting the volume of prefused ${\mathrm{CaCl}}_{2}$ emulsion from that of the corresponding postfused droplet. For the postfused droplet, the original volume was calculated from the observed two-dimensional geometry by assuming the streamwise-axisymmetric three-dimensional shape. From each volume of the prefused NaAlg aqueous droplet, the prefused ${\mathrm{CaCl}}_{2}$ emulsion, and the postfused droplet, we estimated each reduced radius, which was defined as the radius of a sphere with the same volume.

For the morphology of the postfused droplet, we checked the front–back asymmetry based on image analysis. We quantified the “asymmetry index” *A* of the droplet as the area ratio between the projected areas of the anterior half ($S_{a}$) and the posterior half ($S_{p}$), which were observed from the centroid of the droplet. We defined the criterion for the front–back symmetric shape as $A=S_{a}/S_{p}\geq 0.91$ and that for the front–back asymmetric shape as $A=S_{a}/S_{p}<0.91.$ In this analysis, only the data points for which the droplet fusion occurred were plotted. Thus, as indicated by the data points in Figure 6a, the droplet fusion occurred only with limited combinations of the radii of a NaAlg aqueous droplet and a ${\mathrm{CaCl}}_{2}$ emulsion. The radius of the prefused NaAlg aqueous droplet $r_{\mathrm{NaAlg}}$ is negatively correlated with that of the prefused ${\mathrm{CaCl}}_{2}$ emulsion $r_{\mathrm{CaCl}}2$. Figure 6b shows the relationship between the radii of fused droplets $r_{F}$ and those of the corresponding prefused ${\mathrm{CaCl}}_{2}$ emulsions $r_{\mathrm{CaCl}}2$. The sizes of the fused droplets were almost constant as ∼35μm in their radii, regardless of the sizes of the ${\mathrm{CaCl}}_{2}$ emulsions. Furthermore, the resultant morphologies clearly depended on the sizes of the ${\mathrm{CaCl}}_{2}$ emulsions. Among the observed fused droplets, only those that underwent fusion with smaller ${\mathrm{CaCl}}_{2}$ emulsions with the radii less than 20μm were deformed into front–back asymmetric shapes.

## 4. Discussion

In this study, we observed the formation of the droplets with characteristic front–back asymmetric shapes through the anisotropic gelation in a confined channel flow. The anisotropic gelation in this system is triggered by the fusion of a NaAlg aqueous droplet and a ${\mathrm{CaCl}}_{2}$ emulsion. Basically, the surfactant monolayer aligned at the oil/water interfaces tends to prevent fusion even when the two interfaces come into contact with each other. However, the rupture of the contacted oil/water interfaces can stochastically occur due to the shear friction and/or thermal fluctuation at a microscopic scale. Stochastic droplet fusion is likely to occur at a stagnation point in the flow field in the organic phase, as the contact duration of a flowing ${\mathrm{CaCl}}_{2}$ emulsion with the NaAlg aqueous phase becomes the longest. In order to clarify the locations of the stagnation points and further discuss the mechanism of the anisotropic gelation in a confined channel flow, we analyzed the flow field inside and around the droplet.

**Figure 7:**
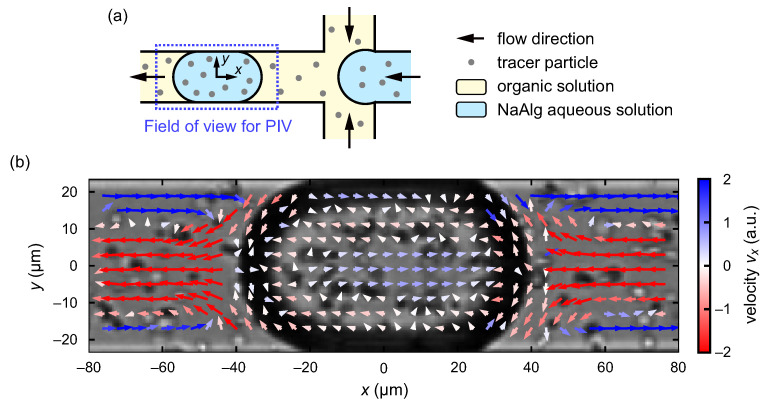
(**a**) Schematic illustration of the field of view for the PIV analysis, depicted by the blue dashed rectangle (not to scale). The droplet frame is adopted and the instantaneous center of mass of a flowing droplet is set as the origin. (**b**) Velocity field in the droplet frame inside and around the droplet flowing just after the junction. Color code corresponds to the velocity component in the *x* direction.

We performed PIV analysis for the tracer particles dispersed in the organic and aqueous phases. We adopted the droplet frame and set the instantaneous center of mass of the droplet as the origin of the two-dimensional coordinates (x, y), as shown in Figure 7a. Figure 7b shows the velocity field inside and around the droplet obtained from PIV. In the NaAlg aqueous phase in the droplet, the flow velocities are relatively small compared with those in the organic phase, since the viscosity of the NaAlg aqueous solution is higher than that of the organic solution in our experiments. The directions of the velocities inside the droplet are slightly backward (blue) at the center of the droplet and slightly forward (red) around it, indicating the existence of a pair of convection rolls. In the present observation by bright-field optical microscopy, the particles near the wall are not clear since oil/water interfaces are visualized as thick dark regions. Hence, the velocity field inside the droplet near the wall, which should steeply change from a backward direction (blue) at the wall boundary to a forward direction (red) in the droplet, might not be precisely extracted.

In contrast, the velocity field outside of the droplet is clearly visualized in this analysis. The backward flow (blue) near the wall boundary and forward flow (red) near the central axis of the channel, as well as their turnover near the droplet surface in the front and back sides, can be observed in the droplet frame, as shown in Figure 7b. In such a flow field in the organic phase, the stagnation points in the present geometry are located on the front and back ends of the droplet surface. Since the velocities in the NaAlg aqueous phase are much smaller than those in the organic phase, the stagnation point in the organic phase determines contact duration. In addition, the stagnation point at the front end, not that at the back end, is the most important since a ${\mathrm{CaCl}}_{2}$ emulsion tends to fuse into the NaAlg aqueous phase just before the breakup into the droplet. Therefore, the fusion events of the NaAlg aqueous phase and the ${\mathrm{CaCl}}_{2}$ emulsion will be most likely be located around the front end of the aqueous phase, resulting in front–back asymmetric gelation.

The analyzed flow field inside the droplet was qualitatively consistent with the three-dimensional PIV analysis on a flowing droplet confined in a similar geometry [50], where the backward flow near the four sidewalls and the forward flow near the corners were observed. The stagnation points on the droplet surface were reported to be located at the tip of the droplet and near the sidewall, but micrometer-sized ${\mathrm{CaCl}}_{2}$ emulsions would not approach those close to the sidewall due to geometrical reasons. It is notable that in the present study, we also visualized two-dimensional flow in the surrounding organic phase. In view of the flow both in the aqueous and organic phases, the only stagnation point around the droplet surface is located at the tip of the droplet.

In summary, we fabricated the micrometer-sized droplets with front–back asymmetric shapes using droplet-based microfluidics. As a simple strategy to fabricate the asymmetric droplets, we employed the fusion of the NaAlg aqueous phase with a ${\mathrm{CaCl}}_{2}$ emulsion at the junction designed in a microchannel. The fusion was likely to occur around the tip of the NaAlg aqueous phase, which would be a stagnation point of the flow field of the organic phase, resulting in anisotropic gelation and, thus, the formation of the front–back asymmetric droplets. Since the anisotropically gelated droplets kept their shape even outside of the narrow channel, the proposed mechanism will be potentially applicable as a novel fabrication technique of microparticles with asymmetric shapes.

Further improvements in the controllability of the fusion event in a periodic droplet generation, such as the controls of the timing and location of the fusion, e.g., by stimulation with a pulse electric field [51] and/or by channel design [32], will be interesting future studies. To finely tune the shape of the fused droplet, the correlation of the shape with relevant experimental parameters such as surface tension, viscosities, etc., should be further clarified. Synthesizing microparticles with front–back asymmetry and studying the flow around them will result in deeper understanding of the transport phenomena of artificial and biological suspensions under confinements, such as blood cells or functional capsules in blood flow [52,53] and biological polar microswimmers moving near the wall [54].

## Data Availability

The data presented in this study are available upon reasonable request from the corresponding author.

## Supplementary Materials

The followings are available online at https://www.mdpi.com/article/10.3390/mi12091121/s1, Video S1: Generation of a droplet with a symmetric shape, Video S2: Generation of a droplet with an asymmetric shape, Video S3: NaAlg aqueous droplet with a symmetric shape, Video S4: Fabricated fused droplet with a front–back asymmetric shape, which is kept even outside the narrow microchannel.

## Abbreviations

The following abbreviations are used in this manuscript:

PEG	Poly(ethylene glycol);
PLGA	Poly(lactic-co-glycolic acid);
UV	Ultraviolet;
${\mathrm{CaCl}}_{2}$	Calcium chloride;
NaAlg	Sodium alginate;
PIV	Particle image velocimetry;
PDMS	Poly(dimethylsiloxane);
PTFE	Poly(tetrafluoroethylene);
NA	Numerical aperture.
